# Remdesivir as a potent antiviral against prototype and current epidemic Oropouche virus strains (BeAn19991 and PE-IAM4637)

**DOI:** 10.1016/j.virusres.2025.199680

**Published:** 2025-12-19

**Authors:** Rokusuke Yoshikawa, Yoshiyasu Ishii, Naomi Sano, Jiro Yasuda

**Affiliations:** a Department of Emerging Infectious Diseases, Institute of Tropical Medicine (NEKKEN), Nagasaki University, Nagasaki, Japan; b Department of Emerging Infectious Diseases, National Research Center for the Control and Prevention of Infectious Diseases (CCPID), Nagasaki University, Nagasaki, Japan; c School of Tropical Medicine and Global Health, Nagasaki University, Nagasaki, Japan; d Graduate School of Biomedical Sciences, Nagasaki University, Nagasaki, Japan; e Brazil Research Station, National Research Center for the Control and Prevention of Infectious Diseases (CCPID), Nagasaki University, Nagasaki, Japan

**Keywords:** Oropouche virus, Reporter virus, Antiviral drug, Remdesivir

## Abstract

The Oropouche virus (OROV), an orthobunyavirus transmitted by biting midges, is the causative agent of Oropouche fever, which has caused multiple outbreaks in South and Central America. During the most recent epidemic in 2023–2025, >25,000 laboratory-confirmed cases were reported in Brazil, and no licensed antivirals have been reported to be effective date. In this study, we generated a recombinant OROV-expressing enhanced green fluorescent protein (rOROV/GFP) to facilitate rapid and sensitive antiviral evaluation. Growth kinetics demonstrated that rOROV/GFP replicated less efficiently than wild-type rOROV and that the historical prototype strain (BeAn19991) exhibited higher replication efficiency than the recent epidemic isolate (PE-IAM4637) in both Vero E6 and Huh7 cells. Using this system, we evaluated the antiviral activity of ribavirin, favipiravir (T-705), and remdesivir against OROV. All three compounds inhibited OROV replication in a dose-dependent manner, with remdesivir showing the greatest potency (IC₅₀ values of 0.31 µM). Taken together, our findings highlight remdesivir as a promising candidate for the treatment of Oropouche fever caused by OROV. Furthermore, we established rOROV/GFP as a powerful tool for antiviral drug screening.

## Highlights

• We generated a recombinant reporter OROV that expresses the eGFP fluorescent protein in infected cells.
• We found that remdesivir efficiently inhibited the replication of Oropouche virus (OROV) using this reporter OROV.
• We demonstrated strain-dependent differences in the replication efficiency of OROV.

The Oropouche virus (OROV), an orthobunyavirus of the family *Peribunyaviridae* transmitted by biting midges, was first isolated in Trinidad and Tobago in 1955 (Anderson et al., 1961). Since then, OROV has caused >80 outbreaks in South and Central America (Srivastava et al., 2025). Oropouche virus disease (Oropouche fever) is an acute febrile illness that typically presents with fever, arthralgia, myalgia, retroorbital pain, and photophobia. During the most recent outbreak in 2023–2025, over 25,000 laboratory-confirmed cases were reported, predominantly in Brazil, with nine documented fatalities (Bandeira et al., 2024; Gangwar et al., 2025; Ministry of Health in Brazil, 2025). Currently, there are no licensed antiviral therapies available for OROV infections.

Historically, viral RNA polymerases have been attractive targets for the development (Fig. 3) of antiviral drugs. Prominent examples include HIV-1 reverse transcriptase (HIV-1 RT), hepatitis B virus polymerase (HBV pol), and hepatitis C virus replicase (NS5B), all of which are the targets of clinically approved antivirals (Neogi et al., 2020). Most current first-line antiviral regimens rely on nucleoside analogs directed against these enzymes (Neogi et al., 2020). Ribavirin is licensed as a broad-spectrum antiviral agent (Neogi et al., 2020). Other nucleoside analogs, such as favipiravir (T-705) and remdesivir, also demonstrate broad-spectrum activity against several highly pathogenic RNA viruses, including the severe acute respiratory syndrome coronavirus 2 (SARS-CoV-2) and the Ebola virus (Neogi et al., 2020; Tchesnokov et al., 2019).

Given the absence of approved therapeutics for OROV and the demonstrated potential of nucleoside analogs as broad-spectrum antivirals, experimental systems that allow the efficient assessment of candidate compounds are urgently needed. Reporter viruses provide such a platform, enabling the direct monitoring of viral replication and quantitative evaluation of antiviral activity. In this *Short Communication*, we described the generation of an OROV reporter and its application in evaluating the antiviral efficacy of three nucleoside analogs: ribavirin, favipiravir (T-705), and remdesivir.

**Fig. 1:**
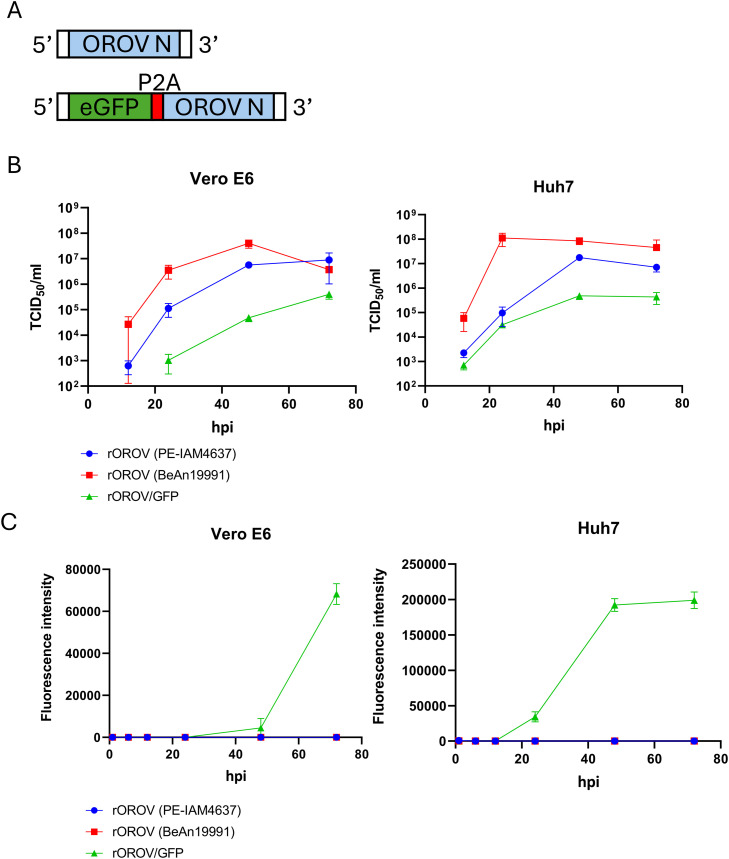
Rescue and characterization of rOROV/GFP. (A) Schematic representation of the wild-type rOROV S segment and the reporter rOROV S segment engineered to express eGFP and N proteins separated by a porcine teschovirus-1 2A self-cleaving peptide (P2A). (B) Growth kinetics of rOROV (PE-IAM4637), rOROV (BeAn19991), and rOROV/GFP in Vero E6 and Huh7 cells at an MOI of 0.1. Viral titers were determined at 12, 24, 48, and 72 hpi and shown as TCID₅₀/mL. (C) GFP fluorescence intensity in infected cells was measured using a microplate reader (SpectraMax iD5: Moleculardevices) at 12, 24, 48, and 72 hpi. Fluorescence values were normalized to background signals from uninfected control wells. The blue dots (●), red squares (■), and green triangles (▲) represent rOROV (PE-IAM4637), rOROV (BeAn19991), and rOROV/GFP, respectively. Error bars indicate standard deviations (*n* = 3).

First, we generated recombinant OROVs (rOROVs) using two strains: the epidemic isolate OROV PE-IAM4637 (obtained from a human case of Oropouche fever in Brazil in 2024; Azevedo et al., 2024) (accession number: PQ073181-PQ073183) and the prototype strain OROV BeAn19991 (originally isolated from a sloth in Brazil in 1960; Tilston-Lunel et al., 2015) (accession number: KP052850-KP052852). The rationale for selecting PE-IAM4637 was that it had been isolated from a patient during the 2024 outbreak, its nearly full-length genome had been identified from the actual virus isolate, and its sequence had already been accepted for publication at the time this study was initiated (2024). However, because the untranslated regions (UTRs) of PE-IAM4637 were incomplete and the viral isolate was no longer available, we reconstructed the missing sequences by comparing UTRs from multiple OROV strains, including BeAn19991. Sequence alignments were performed using MUSCLE, and conserved regions across strains were identified. Based on these conserved sequences, the incomplete UTRs of PE-IAM4637 were complemented to generate full-length genomic segments for the rescue of recombinant viruses. Consequently, in the reconstructed system, the UTR sequences of all genomic segments of PE-IAM4637 and BeAn19991 were identical. Full-length cDNAs of each genomic segment (L, M, and S) of the OROV genome were synthesized (Eurofins, Japan). Each genomic segment (L, M, and S) was amplified and cloned into a T7 vector between the T7 promoter and a hepatitis delta virus ribozyme sequence to generate six plasmids (pT7-L[PE], pT7-M[PE], pT7-S[PE], pT7-L[BeAn], pT7-M[BeAn], and pT7-S[BeAn]). Cloning was performed using an In-Fusion HD Cloning Kit (Takara, Japan). In addition, the open reading frame (ORF) encoding the viral RNA-dependent RNA polymerase (RdRP) and the nucleocapsid protein (N) of OROV(PE) were cloned into the pCAGGS vector using an In-Fusion HD Cloning kit to prepare pCAGGS-RdRp and pCAGGS-N. To rescue each rOROV, BHK-T7/9 cells, a hamster kidney–derived BHK cell clone stably expressing T7 RNA polymerase (Ito et al., 2003; Macpherson et al., 1962), were co-transfected with pCAGGS-RdRp (0.5 μg), pCAGGS-N (0.5 μg), pT7-L (1.5 μg), pT7-M (1 μg), and pT7-S (1.5 μg) using LT-1 (Mirus). Eight days after transfection, the virus-containing supernatant was transferred to Vero E6 cells and incubated at 37 °C for 4 days. Next, to generate rOROV-encoding eGFP, the porcine teschovirus-1 2A peptide linker (P2A) was inserted between the eGFP and OROV N ORF within pT7-S[PE] to prepare pT7-S[eGFP] (Fig. 1A). The transfection of pT7-S[eGFP] with pT7-M[PE], pT7-L[PE], pCAGGS-RdRp, or pCAGGS-N did not rescue rOROV/GFP. In contrast, when pT7-S[eGFP] was co-transfected into BHK-T7/9 cells along with pT7-M[BeAn], pT7-L[BeAn], pCAGGS-RdRp, and pCAGGS-N, successful recovery of rOROV/GFP was achieved. To compare the growth kinetics of rOROV and rOROV/GFP, Vero E6 cells (ATCC CRL-1586) derived from the kidney of an African green monkey and Huh7 cells (JCRB Cell Bank, JCRB0403) derived from the liver of humans were infected with 10^4.22^ TCID_50_ of rOROV (PE-IAM4637), rOROV (BeAn19991), or rOROV/GFP. Both the cell lines are susceptible to OROV infection (Tilston-Lunel et al., 2015; Scachetti et al., 2025). The target cells were seeded in 12-well plates at 10^5^ cells per well. Each OROV was inoculated into each well, and the plates were incubated for 1 h at 37 °C for viral adsorption. Culture supernatants were collected at 12, 24, 48, and 72 h post-infection (hpi), and viral titers were determined using the TCID_50_ (50 % tissue culture infectious dose) assay (Gunter et al., 2024). The maximum titers of rOROV (PE-IAM4637), rOROV (BeAn19991), and rOROV/GFP produced in Vero E6 cells were 8 × 10^6^ (72 hpi), 4 × 10^7^ (48 hpi), and 4 × 10^5^ (72 hpi) TCID_50_/ml, respectively (Fig. 1B). The maximum titers of rOROV (PE-IAM4637), rOROV (BeAn19991), or rOROV/GFP produced in Huh7 cells were 1.8 × 10^7^ (48 hpi), 1.1 × 10^8^ (24 hpi), or 4.8 × 10^5^ (48 hpi) TCID_50_/ml, respectively (Fig. 1B). These results indicated that the replication efficiency of rOROV/GFP was lower than that of rOROV (PE-IAM4637) and rOROV (BeAn19991). Furthermore, rOROV (BeAn19991) exhibited a higher replication efficiency than rOROV (PE-IAM4637) in both Vero E6 and Huh7 cells. Additionally, all rOROV showed greater sensitivity to Huh7 cells than Vero E6 cells. Similar observations have been reported for other arboviruses, such as the Zika virus, which also replicates more efficiently in Huh7 cells than in Vero E6 cells (Vicenti I et al., 2018). Although the underlying mechanism remains unclear, this difference likely reflects hepatocyte-specific characteristics, such as abundant lipid metabolism and membrane trafficking pathways that facilitate viral assembly. Previous studies have shown that hepatoma-derived cells utilize Golgi complex remodeling and ESCRT-mediated budding for efficient particle formation, and OROV similarly exploits the Golgi apparatus and recruits ESCRT components during its replication cycle (Mankouri et al., 2016; Inoue et al., 2018). Furthermore, lipid metabolism and lipophagy have been implicated in supporting arbovirus replication in Huh7 cells (Qin et al., 2022; Shen et al., 2023; Barbosa et al., 2018). Future studies comparing the transcriptomic profiles of these cell lines may help identify novel host determinants critical for OROV infection.

**Fig. 2:**
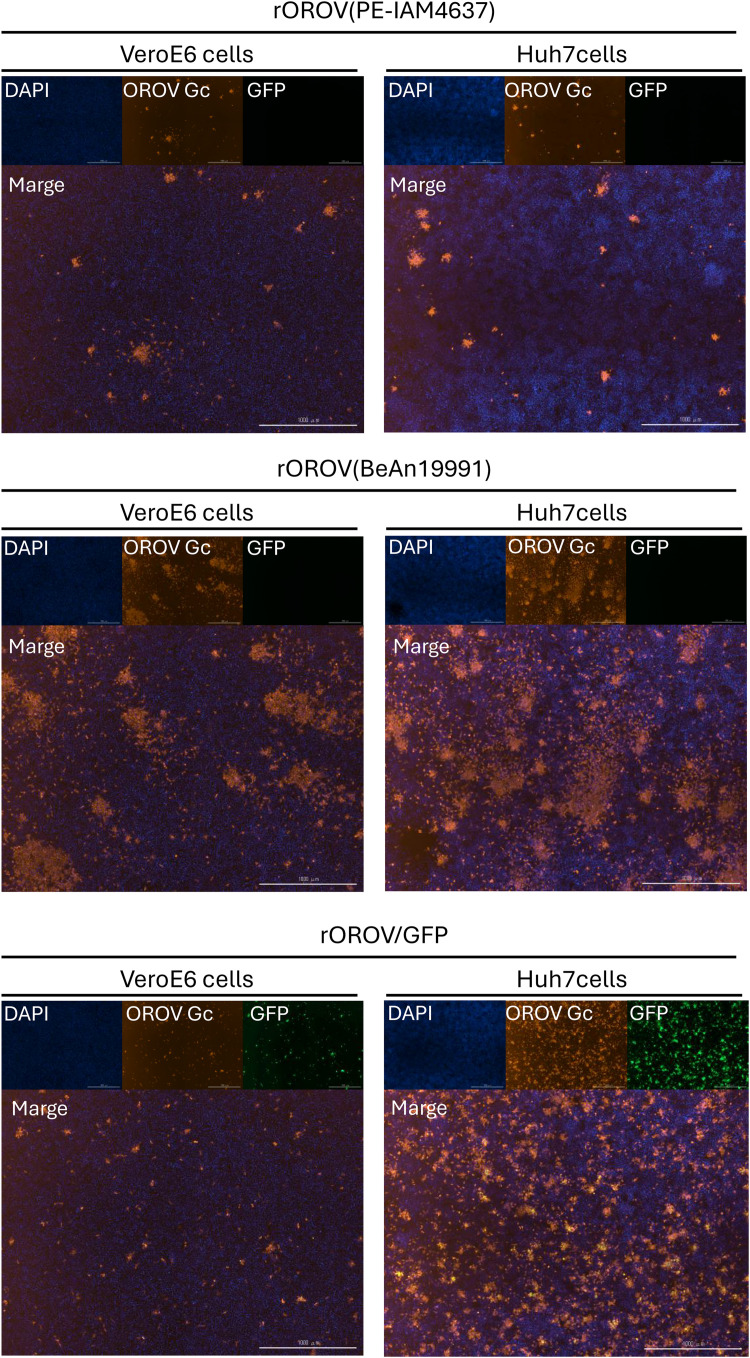
Expression of GFP and OROV Gc proteins. Fluorescence microscopy images of the Vero E6 and Huh7 cells infected with rOROV (PE-IAM4637), rOROV (BeAn19991), or rOROV/GFP, acquired by using SpectraMax iD5 (Moleculardevices) at 24 hpi. GFP expression was visualized directly, while OROV Gc proteins were detected by indirect immunofluorescence using an anti-OROV Gc antibody, followed by a fluorescent secondary antibody. Nuclei were counterstained with DAPI. Representative fields from three independent experiments are shown.

Next, we quantified green fluorescence intensity in Vero E6 and Huh7 cells infected with rOROV/GFP. As shown in Fig. 1C, the fluorescence intensity of Huh7 cells reached a plateau at 48 hpi, whereas that of Vero E6 cells did not reach a plateau within 72 hpi. Consistent with the TCID assay results, rOROV/GFP exhibited faster growth kinetics in Huh7 cells than in Vero E6 cells. Additionally, at 24 hpi, eGFP expression was observed in all cells expressing OROV Gc glycoprotein (Fig. 2). Huh7 cells are more susceptible to OROV infection than Vero E6 cells and are derived from the human liver tissue. Therefore, Huh7 cells were used to evaluate the antiviral efficacy of the compounds.

**Fig. 3:**
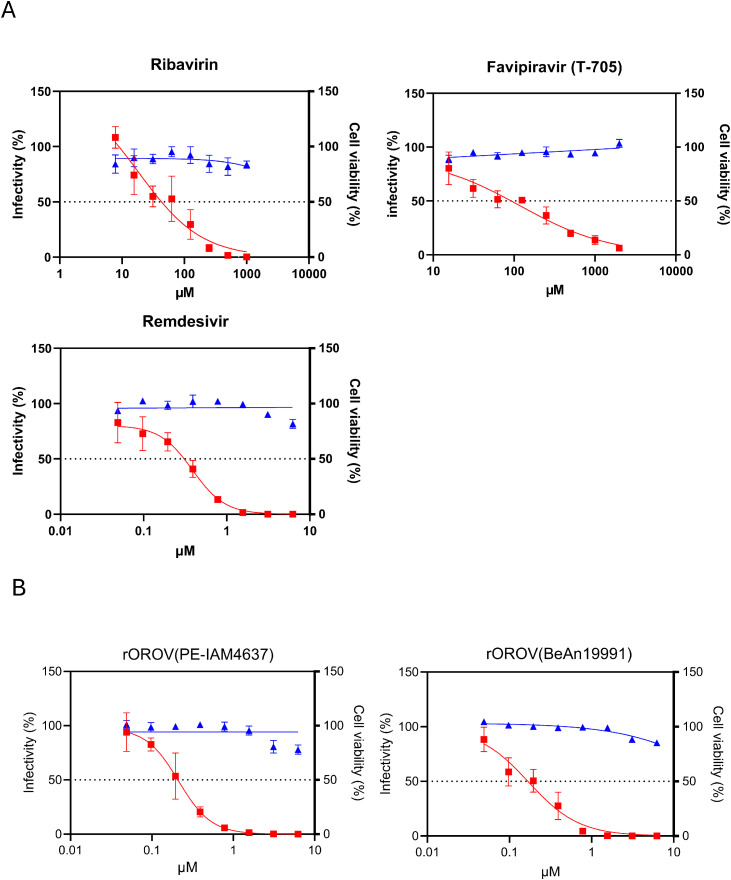
Antiviral effect of nucleoside analogs against OROV in Huh7 cells. Huh7 cells were infected with (A) rOROV/GFP, (B) rOROV (PE-IAM4637), or rOROV (BeAn19991) in the presence of the indicated concentrations of ribavirin, favipiravir (T-705), or remdesivir. After 24 hpi, cells were fixed and stained with DAPI to visualize nuclei. (A) GFP expression or (B) OROV Gc antigen staining was used to identify infected cells. Images were acquired by fluorescence microscopy. Infectivity and cell viability were calculated by counting the number of infected cells and the total nuclei number, respectively. The percent of infectivity and cell viability in DMSO-treated cells was set as 100 %. The red squares (■), and blue triangles (▲) represent the percent of infectivity and cell viability, respectively. Data are normalized to mean values for untreated control and presented as mean ± SEM from three biological replicates (*n* = 3).

To evaluate the antiviral activity of ribavirin (Selleck Biotechnology), favipiravir (T-705) (Selleck Biotechnology), and remdesivir (Cayman Chemical), Huh7 cells were infected with rOROV/GFP (10^2.8^ TCID_50_), and each compound was added at 1 hpi. After 24 h, the cells were fixed with 4 % paraformaldehyde for 1 h, and the nuclei were stained with 4′,6-diamidino-2-phenylindole (DAPI; Roche). Images were acquired using a Cytation 5 Imaging Plate Reader (Agilent Technologies). The number of cell nuclei and infected cells was quantified using CellProfiler software with a customized analysis pipeline (Sakurai et al., 2018; Imamura et al., 2021). All the compounds reduced rOROV/GFP infectivity in a dose-dependent manner (Fig. 3A). Under these conditions, no cytotoxicity was observed (Fig. 3A and B). Among the tested compounds, remdesivir showed the highest antiviral potency, with the lowest IC₅₀ (50 % inhibitory concentration) value (0.31 µM), compared with ribavirin (10.5 µM) and favipiravir (90.9 µM) (Fig. 3A). To evaluate the antiviral activity of remdesivir against rOROV (PE-IAM4637) and rOROV (BeAn19991), Huh7 cells were infected with either of the virus strains, and subsequently treated with remdesivir. The number of infected cells was quantified by indirect immunofluorescence using an anti-OROV Gc antibody (ATCC V-505–701–562). Consistent with the results obtained for rOROV/GFP, remdesivir reduced the infectivity of rOROV (PE-IAM4637) and rOROV (BeAn19991) in a dose-dependent manner, with IC₅₀ values of 0.21 and 0.17 µM, respectively (Fig. 3B). The phylogenetic analysis of the OROV L segment encoding RdRp has shown that current epidemic strains, including PE-IAM4637, form one cluster, whereas the prototype strain BeAn19991 belongs to another genetically distinct cluster (Scachetti et al., 2025). Therefore, it is reasonable to hypothesize that remdesivir could be effective against most of the OROV strains currently circulating. However, further experimental validation using additional isolates is warranted.

The generation of a GFP-expressing OROV is a powerful tool for the rapid and sensitive evaluation of antiviral compounds. Our results indicated that ribavirin, favipiravir (T-705), and remdesivir can inhibit OROV infection, with remdesivir showing the highest potency. In comparative experiments evaluating the antiviral efficacy of these three drugs against SARS-CoV-2, remdesivir exhibited the highest activity (Wang et al., 2020). The superiority of remdesivir may be partially explained by its chemical design as a phosphoramidate prodrug. Unlike ribavirin and favipiravir, which require multiple intracellular phosphorylation steps and compete with host nucleotide pools, remdesivir bypasses the first rate-limiting phosphorylation step (Bigley et al., 2020; Li et al., 2021). Therefore, remdesivir confers greater efficiency in cellular uptake and enhanced stability than other compounds. To date, the mechanism by which remdesivir exerts its antiviral activity against OROV remains unclear. Nevertheless, its active metabolite, GS-441,524, has been reported to inhibit SARS-CoV-2 replication through incorporation into the viral RdRp, permitting the extension of the RNA chain by three nucleotides before inducing delayed chain termination (Gordon, 2022). Although direct biochemical data on OROV RdRp are lacking, it is plausible that remdesivir suppresses OROV infection through a similar mechanism.

In this study, we observed that the replication efficiency of the OROV strain PE-IAM4637 (an epidemic isolate in 2024) was lower than that of the historical prototype strain BeAn19991. Interestingly, although rOROV/GFP could not be rescued using all the segments from PE-IAM4637, it was successfully generated when the S segment from PE-IAM4637 and the M and L segments from BeAn19991 were used. These findings suggest that differences in the M and L segments may contribute to the reduced replication efficiency of OROV strain PE-IAM4637. The PE-IAM4637 sequence used in this study was obtained from a virus isolated directly from a human patient during the 2024 outbreak. In contrast, the BeAn19991 sequence was derived from the virus that had undergone extensive passaging in various cell lines (Acrani et al, 2015), suggesting that the BeAn19991 strain may already be adapted to grow efficiently in cultured cells. In fact, the L segment sequence of the BeAn19991 strain has been submitted twice, in 2003 and 2015 (NC_005776.1 and KP052850.1), and it has been reported that these sequences differ from each other (Acrani et al., 2015). Such discrepancies may reflect sequence changes that occurred during cell culture passaging over time. In this study, we adopted the sequence submitted in 2015 for the construction of recombinant OROV (BeAn19991). Previous studies have shown that arboviruses can undergo cell culture-specific adaptations that influence replication kinetics and host interactions (Ciota et al, 2010). Consistent with our findings, Fischer et al. reported that the OROV-CUB2024 strain, isolated from the serum of an Italian tourist returning from Cuba in 2024, exhibited lower replication efficiency in Vero E6 cells compared to the prototype strain OROV-PT1960 (Fischer et al., 2025). In contrast, the 2023–2024 epidemic isolate AM0088, which is distinct from PE-IAM4637, has been reported to replicate more efficiently in Huh7 cells than in BeAn19991 (Scachetti et al., 2025). These observations suggest that at least two distinct phenotypes—one with low replication efficiency and one with high replication efficiency—exist among OROV strains circulating in Brazil during 2023–2024. Phylogenetic analysis revealed that PE-IAM4637 and AM0088 belong to the same cluster, whereas BeAn19991 forms a distinct cluster (data not shown) (Naveca et al., 2024; Scachetti et al., 2025). These findings suggest that the overall genetic distance may not be the primary determinant of differences in viral replication kinetics. To further investigate it, we compared the amino acid sequences of individual viral proteins between PE-IAM4637 and AM0088. The N and the nonstructural protein (NSs) were found to be identical. In contrast, six amino acid substitutions were identified in the glycoprotein (GP) and one in the RdRp (Table 1). These differences may contribute to the distinct replication phenotypes observed among OROV strains.

**Table 1:** Comparison of amino acid sequences between PE-IAM4637 and AM0088.

GP	position	22	176	552	812	846	1068
	PE-IAM4637	Ser	Ile	Ser	Ser	Ile	Ala
	AM0088	Asn	Val	Ala	Pro	Val	Val
RdRp	position	239	851	1361	1980	2194	
	PE-IAM4637	Lys	Lys	Ile	Leu	Glu	
	AM0088	X	X	X	Met	X	
X: not identified							

The discrepancies between our study and previous studies may also be explained by the differences in methodology. For quantification of progeny virus production, our study and a previous study by Scachetti *et al*. measured infectious viral titers in culture supernatants, whereas Fischer et al. quantified viral RNA levels in culture supernatants. In addition, the timing of supernatant collection differed among studies, which may explain differences in replication kinetics between strains. It is also important to note that our study utilized recombinant viruses, whereas Fischer et al. and Scachetti et al. used clinical isolates. These methodological differences highlight the need for future comparative studies under standardized experimental conditions to more accurately assess replication kinetics across multiple OROV strains.

There are some additional limitations in this study. First, the mechanism of remdesivir’s antiviral activity against OROV remains speculative. Further studies, such as in vitro polymerase assays using purified OROV RdRp, would be required to confirm the proposed mechanism. Second, the reconstructed UTRs of PE-IAM4637 were inferred from the conserved sequences across multiple strains due to the lack of a complete viral isolate, which might affect replication efficiency and regulatory functions.

Taken together, our findings suggest that remdesivir has the potential to serve as an effective treatment for Oropouche fever. Additionally, the rOROV/GFP represents a valuable tool for antiviral drug screening.

## Funding

This research was supported by the Moonshot Research & Development Program from the 10.13039/501100002241Japan Science and Technology Agency (JST) under Grant Number JPMJMS2025 (J.Y.); grants from the 10.13039/100009619Japan Agency for Medical Research and Development (AMED) under Grant Numbers JP25wm0125011, JP25fm0208101, JP243fa627004 (J.Y.), and JP24gm1610007 (R.Y); and the 10.13039/501100001691Japan Society for the Promotion of Science under Grant Number 25K11741 (R.Y.).

## Data availability

Data will be made available on request.

## CRediT authorship contribution statement

**Rokusuke Yoshikawa:** Writing – review & editing, Writing – original draft, Visualization, Validation, Resources, Methodology, Investigation, Funding acquisition, Formal analysis, Data curation, Conceptualization. **Yoshiyasu Ishii:** Writing – review & editing, Visualization, Validation, Methodology, Investigation, Formal analysis. **Naomi Sano:** Investigation. **Jiro Yasuda:** Writing – review & editing, Validation, Supervision, Project administration, Funding acquisition, Conceptualization.

## Declaration of competing interest

The authors declare that they have no competing financial interests or personal relationships that may have influenced the work reported in this study.
